# Identification of differentially expressed genes associated with semigamy in Pima cotton (*Gossypium barbadense *L.) through comparative microarray analysis

**DOI:** 10.1186/1471-2229-11-49

**Published:** 2011-03-16

**Authors:** Jessica Curtiss, Laura Rodriguez-Uribe, J McD Stewart, Jinfa Zhang

**Affiliations:** 1 Department of Plant and Environmental Sciences, New Mexico State University, Las Cruces, NM 88003, USA; 2 Department of Crop, Soil, and Environmental Sciences, University of Arkansas, Fayetteville, AR 72701, USA

## Abstract

**Background:**

Semigamy in cotton is a type of facultative apomixis controlled by an incompletely dominant autosomal gene (Se). During semigamy, the sperm and egg cells undergo cellular fusion, but the sperm and egg nucleus fail to fuse in the embryo sac, giving rise to diploid, haploid, or chimeric embryos composed of sectors of paternal and maternal origin. In this study we sought to identify differentially expressed genes related to the semigamy genotype by implementing a comparative microarray analysis of anthers and ovules between a non-semigametic Pima S-1 cotton and its doubled haploid natural isogenic mutant semigametic 57-4. Selected differentially expressed genes identified by the microarray results were then confirmed using quantitative reverse transcription PCR (qRT-PCR).

**Results:**

The comparative analysis between isogenic 57-4 and Pima S-1 identified 284 genes in anthers and 1,864 genes in ovules as being differentially expressed in the semigametic genotype 57-4. Based on gene functions, 127 differentially expressed genes were common to both semigametic anthers and ovules, with 115 being consistently differentially expressed in both tissues. Nine of those genes were selected for qRT-PCR analysis, seven of which were confirmed. Furthermore, several well characterized metabolic pathways including glycolysis/gluconeogenesis, carbon fixation in photosynthetic organisms, sesquiterpenoid biosynthesis, and the biosynthesis of and response to plant hormones were shown to be affected by differentially expressed genes in the semigametic tissues.

**Conclusion:**

As the first report using microarray analysis, several important metabolic pathways affected by differentially expressed genes in the semigametic cotton genotype have been identified and described in detail. While these genes are unlikely to be the semigamy gene itself, the effects associated with expression changes in those genes do mimic phenotypic traits observed in semigametic plants. A more in-depth analysis of semigamy is necessary to understand its expression and regulation at the genetic and molecular level.

## Background

Semigamy is a naturally occurring mutation that conditions atypical reproductive behavior in plants. It has been described in 13 plant species including *Rudbeckia *spp., *Zephyranthes *spp., *Cooperia pedunculata*, *Coix aquatica*, *Gossypium barbadense*, and most recently *Theobroma cacao *[1-6]. During semigamy, the sperm and egg cells undergo syngamy or cellular fusion, but forgo karyogamy, the fusion of the sperm and egg nuclei. In most semigametic plant species, the male nucleus and its derivatives are sequestered following syngamy and do not contribute to the genetic makeup of the zygote [3,4]. However, in *G. barbadense *and *T. cacao*, both of which are members of the plant family Malvaceae, the mode of semigamy is unique in that the male nucleus is not sequestered and does contribute its genetic material to the embryo [5,6]. Consequently, the maternal and paternal nuclei proceed to divide independently resulting in several possible progenies including normal tetraploids, diploids, haploids, or chimeric embryos.

In cotton, semigamy was first observed by Turcotte and Feaster [5] through recovery of a doubled haploid mutant 57-4 from a commercial non-semigametic Pima S-1, which produced haploids at a high frequency, ranging from 25 to 61% when self pollinated. Subsequent breeding and genetic experiments revealed that semigamy was an inheritable trait and controlled by a single incompletely dominant gene, denoted *Se *[7,8]. A unique feature of semigamy in cotton is that expression of the trait in terms of haploid production is controlled by the genotype of both male and female gametes [8]. Zhang and Stewart [8] reported that the semigametic line 57-4 produced 44% haploids when both gametes carried the semigametic gene Se by self pollination, but produced only 11% haploids when crossed as female to its nonsemigametic isoline Pima S-1. However, no haploids were detected when 57-4 was crossed as male to Pima S-1. This indicates that a special microenvironment in the embryo sac provided by the semigametic genotype is essential for haploid production. Also, a similar condition in male gametes with the semigametic genotype can substantially facilitate semigamy expression, indicating that the semigametic gene is expressed in both male and female gametes for a maximum haploid production. This also lays the foundation for searching for the expressed Se gene and associated gene expression using both male and female tissues in the present study.

While there have been attempts at molecular analysis related to semigamy in cotton [9], there is currently little known about the molecular genetics and gene expression of semigamy. Therefore, the objective of this study was to identify differentially expressed genes associated with the semigametic genotype using microarray analysis in order to gain insight into the underlying molecular mechanism of semigamy in cotton. To our knowledge, this is the first report of microarray and quantitative reverse transcription PCR (qRT-PCR) usage associated with semigamy and will hopefully lay the groundwork towards understanding its genetic mechanism, regulation and control.

## Results

### Microarray and data analysis

In this study, RNA from anthers and ovules of flowers at the 0 day post-anthesis (DPA) were extracted from both semigametic mutant 57-4 and its nonsemigametic natural isoline Pima S-1 and compared for transcriptome analysis using Affymetrix GeneChip Cotton Genome Array. The data were submitted to the GEO repository with the series entry number GSE27242 http://www.ncbi.nlm.nih.gov/geo/query/acc.cgi?acc=GSE27242. 284 genes in anthers and 1,864 genes in ovules were found to be differentially expressed in the semigametic genotype 57-4 compared to its non-semigametic isogenic line Pima S-1 (Additional file 1 and 2). Of the 284 differentially expressed genes identified in the semigametic anther tissue, 52 were up-regulated and 232 were down-regulated, while in semigametic ovule tissues 149 genes were up-regulated and 1,678 genes were down-regulated. Since it is known that fewer genes are expressed in male gametes of plants [10], it is not surprising to see much few differentially expressed (DE) genes were identified when anthers were used. Because the *Se *gene appears to be expressed in both male and female gametophytes for maximum haploid production [8], both ovules and anthers were harvested for identifying genes that were consistently up- or down- regulated in both tissues. Out of the 2,067 total differentially expressed genes identified, 127 genes were found to be differentially expressed in both tissues, 115 of which were consistently differentially expressed, i.e., either up- or down- regulated, in both tissues (Additional file 3), which accounted for more than 40% of the DE genes identified in the anthers. For example, among 81 genes with the same GeneBank accession numbers in both tissues, most genes (77) were consistently down-regulated in both anther and ovule tissues of 57-4 and two genes were consistently up-regulated, while only two differentially expressed genes were inconsistent (i.e., up-regulated in one tissue, but down-regulated in another, or *vice versa*). The correlation of the log2 ratios between the two tissues based on the 81 genes was found to be highly significant (r = 0.51, P < 0.01). The common differentially expressed genes identified in both tissues indicates common gene regulation mechanism in different tissues by the semigamy gene in cotton. It also demonstrated the reliability of the microarray technology used in the current study and also provided a greater confidence in our research results.

**Figure 1:**
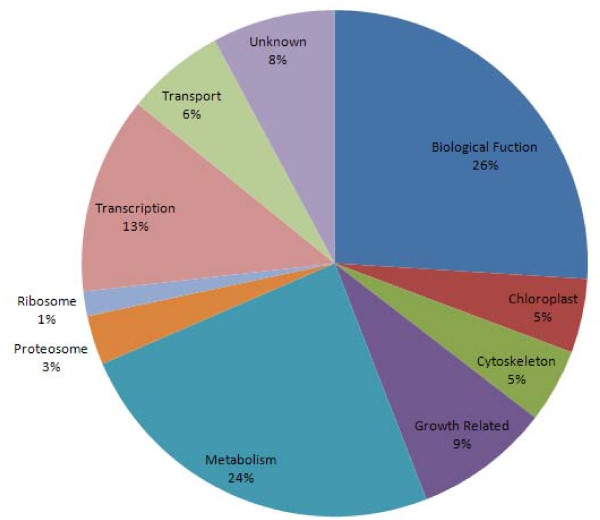
**Distribution of commonly differentially expressed genes in semigametic anthers and ovules based on cellular function**.

The 127 common differentially expressed genes identified in semigametic anthers and ovules were then categorized based on their cellular function (Figure 1) and literature pertaining to their corresponding metabolic or biological pathways was analyzed. Several well characterized pathways, such as glycolysis/gluconeogenesis, carbon fixation in photosynthetic organisms and the tricarboxylic acid (TCA) cycle, were found to be affected in semigametic tissues (Table 1). Additionally, there were several differentially expressed genes related to hormone biosynthesis and response. Both 12-oxophytodienoate reductase [GeneBank: DT466538], which is involved in the biosynthesis of jasmonates, and the gibberellin response protein DELLA-GAI [GeneBank: DT468888] were found to be up-regulated in semigametic tissues. Conversely, an ethylene-responsive transcription factor [GeneBank: DT047349, AW186839], allene oxide synthase [GeneBank: DT047194] which also participates in jasmonate synthesis, and an auxin/indole acetic acid protein [GeneBank: DW517716, CA992726] were found to be down-regulated in semigametic tissues. In addition, (+)-δ-cadinene synthase [GeneBank: U23206, CO107110], which catalyzes the first step in gossypol synthesis in cotton, was found to be up-regulated in semigametic anthers and ovules. Another common finding was the down-regulation of cytoskeletal proteins, such as α-tubulin [GeneBank: DT052122] and β-tubulin [GeneBank: CO124756, DW516614, DT507015] in semigametic tissues. However, genes homologous to actin were found to be up-regulated in semigametic anthers but down-regulated in semigametic ovules. There were also several genes related to oxidative stress, such as iron superoxide dismutase (SOD) [GeneBank: DQ088821] and Cu/Zn SOD [GeneBank: DQ088818, DQ120514], identified as down-regulated in semigametic tissues.

**Table 1:** Noteworthy differentially expressed genes identified in semigametic tissues.

Category	Gene	Log_2 _Signal Anthers	Log_2 _Signal Ovules	GeneBank ID
Glycolysis and TCA	Fructose-bisphosphate aldolase	2.3	-1.5	CA993106/AI054483

	Succinate dehydrogenase	-1.0	-2.3	DT570098/CO122837

	Phosphoglycerate kinase	-1.3	-2.2	DW481615/DW498822

	Glucose-6-isomerase	-	-1.1	DT456471

	Pyruvate dehydrogenase subunit E1	-	-2.1	DT570955

				

Photosynthesis	Oxygen-evolving enhancer protein	-3.6	-1.3	DT458079/CO093680

	Rubisco small subunit precursor	3.5	-1.1	DN780767/CO496683

	Rubisco activase 1	1.8	-	AF329934

	Rubisco activase 2	1.6	-	DQ233255

	Chlorophyll A/B binding protein	1.1	-1.5	CA992778

	Cytochrome b5	-1.3	-1.3	CO085819/DT047754

	Cytochrome c oxidase	-	3.2	CA993773

				

Metabolism	(+)-δ-cadinene synthase	1.4	1.4	U23205/CO107110

	Phosphoethanolamine N-methyltransferase	-1.1	-1.3	DW225135

				

Cytoskeleton	α-Tubulin	-	-1.5	DT052122

	Actin	1.0	-1.4	DN759693/CO084889

	β-Tubulin 1	-1.0	-1.5	CO124765/DW516614

	β-Tubulin 3	-	-2.1	DT557030

	β-Tubulin 8	-1.1	-	CO124872

	Tubulin	-1.4	-2.4	DW507015

				

Hormone-related	12-oxophytodienoate reductase	1.1	1.2	DT466538

	Allene oxide synthase	-1.2	-	DT047194

	DELLA protein GAI	1.3	1.1	DT468888

	Ethylene-responsive transcription factor 5	-2.5	-2.2	DT047349/AW186839

	Ethylene-responsive transcription factor ERF017	-5.0	-2.2	DT049130

	Auxin/Indole acetic acid protein	-2.0	-2.0	DW517716/CA992726

	Auxin repressed protein	-	-1.1	CO127792

	ACC synthase	-1.2	-	DQ122174

	ACC oxidase	1.0	-	DQ116442

				

SOD-related	FeSOD	-1.9	-1.9	DQ088821

	Cytosol Cu/Zn SOD	-	-1.4	DQ088818

	Chloroplast Cu/Zn SOD	-	-1.1	DQ120514

Dashes designate that the gene was not found to be differentially expressed through microarray analysis

### Quantitative reverse transcription PCR

**Figure 2:**
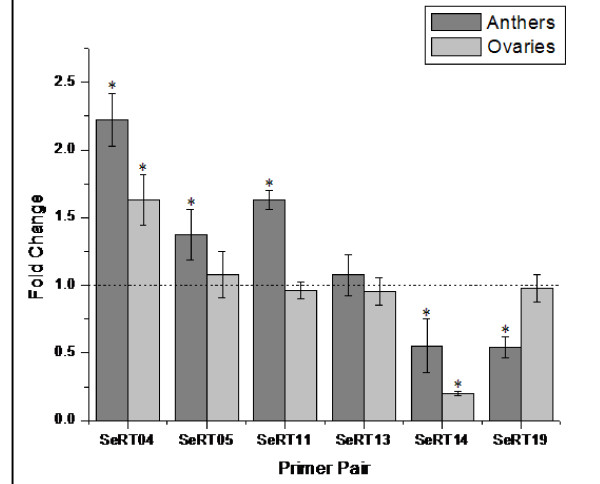
**qRT-PCR results**. SeRT04-Histone H1-III, SeRT05-Transcription initiation factor TFIID, SeRT11-60S acidic ribosomal protein, SeRT13-E3 ubiquitin-protein ligase, SeRT14-High MW heat shock protein, SeRT19-Tubulin beta-8. The dashed line represents gene expression in non-semigametic Pima S-1 (PS-1) tissues. Asterisks (*) indicate that the result was statistically significant between the two genotypes.

Initially, the six most up-regulated and down-regulated genes identified in semigametic tissues by microarray were chosen for confirmation using qRT-PCR (Table 2 and 3). Of the twelve total reactions, seven including transcription initiation factor TFIID (SeRT 05), 60S acidic ribosomal protein P1 (SeRT 11) and β-Tubulin 8 (SeRT 19) in anthers as well as histone H1-III (SeRT 04) and high MW heat shock protein (SeRT 14) in both anthers and ovules, produced significantly different results between the two isogenic genotypes (Figure 2). The statistically significant qRT-PCR results are listed in Table 2.

**Table 2:** Statistically significant qRT-PCR results.

Target Gene	Tissue	qRT-PCR Result	Microarray Result
Histone H1-III	Anthers	2.0-fold increase	6.5-fold increase

Histone H1-III	Ovules	1.6-fold increase	-

β-Tubulin	Anthers	1.8-fold decrease	2.6-fold decrease

High MW heat shock protein	Anthers	1.8-fold decrease	2.8-fold decrease

High MW heat shock protein	Ovules	5.0-fold decrease	12.1-fold decrease

Transcription initiation factor TFIID	Anthers	1.4-fold increase	6.5-fold increase

Rubisco activase 1	Anthers	1.7-fold increase	3.5-fold increase

Rubisco activase 1	Ovules	5.7-fold increase	-

Rubisco activase 2	Anthers	2.3-fold decrease	3.0-fold increase

Rubisco activase 2	Ovules	1.1-fold decrease	-

Rubisco small subunit precursor	Ovules	1.1-fold decrease	2.1-fold decrease

The dashes designate that the gene was not found to be differentially expressed via microarray analysis.

**Table 3:** Results for each gene analyzed using qRT-PCR

Primer Name	Target Gene	Tissue	PS-1 Expression	57-4 Expression
SeRT 04	Histone H1-III	Anthers	1.000 ± 0.119	2.222 ± 0.194
		
		Ovules	1.000 ± 0.081	1.632 ± 0.187

SeRT 05	Transcription initiation factor TFIID	Anthers	1.000 ± 0.158	1.374 ± 0.100
		
		Ovules	1.000 ± 0.149	1.076 ± 0.170

SeRT 11	60S acidic ribosomal protein P1	Anthers	1.000 ± 0.066	1.632 ± 0.073
		
		Ovules	1.000 ± 0.108	0.960 ± 0.061

SeRT 13	E3 ubiquitin-protein ligase	Anthers	1.000 ± 0.143	1.076 ± 0.151
		
		Ovules	1.000 ± 0.077	0.954 ± 0.103

SeRT 14	High MW heat shock protein	Anthers	1.000 ± 0.048	0.552 ± 0.199
		
		Ovules	1.000 ± 0.049	0.201 ± 0.017

SeRT 19	β-Tubulin 8	Anthers	1.000 ± 0.094	0.542 ± 0.077
		
		Ovules	1.000 ± 0.061	0.978 ± 0.099

RBC 01	Rubisco activase 1	Anthers	1.000 ± 0.089	1.674 ± 0.247
		
		Ovules	1.000 ± 0.138	5.745 ± 0.601

RBC 02	Rubisco activase 2	Anthers	1.000 ± 0.027	0.434 ± 0.018
		
		Ovules	1.000 ± 0.017	0.950 ± 0.013

RBC SmSub	Rubisco small subunit precursor	Anthers	1.000 ± 0.074	0.861 ± 0.077
	
		Ovules	1.000 ± 0.026	0.885 ± 0.056

**Figure 3:**
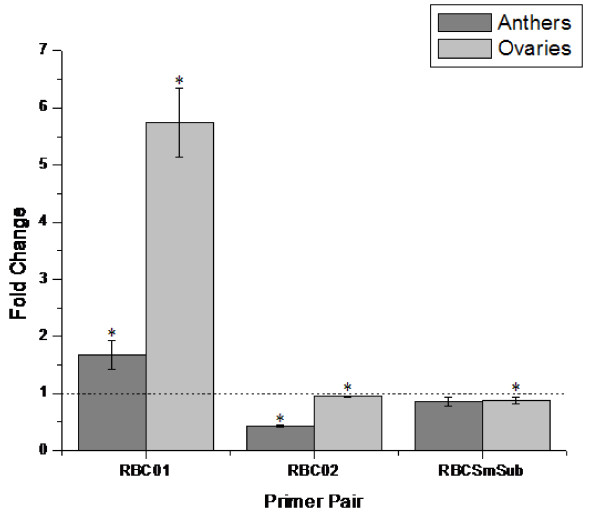
**qRT-PCR results for Rubisco-related target genes**. RBC01-Rubisco activase 1, RBC02-Rubisco activase 2, RBCSmSub-Rubisco smallchain chloroplast precursor. The dashed line represents gene expression in non-semigametic Pima S-1 (PS-1) tissues. Asterisks (*) indicate that the result was statistically significant between the two genotypes.

Previous studies have shown that the rate of photosynthesis, specifically carbon dioxide (CO_2_) fixation, is markedly decreased in semigametic 57-4 cotton plants in comparison to its non-semigametic isoline Pima S-1 [8]. In plants and photosynthetic bacteria, the enzyme Ribulose-1,5-bisphosphate carboxylase/oxygenase (Rubisco) catalyzes the first step in photosynthetic CO_2 _assimilation and is the overall rate limiting step of photosynthesis [11]. As a preliminary probe into any effects of semigamy on the photosynthetic pathways, three differentially expressed Rubisco genes identified via microarray analysis, Rubisco activase 1 [GeneBank: AF329934], Rubisco activase 2 [GeneBank: DQ233255], and a Rubisco small subunit precursor [GeneBank: DN780767], were used to perform six qRT-PCR reactions to study the expression of Rubisco in semigametic versus non-semigametic anther and ovule tissues. The results of the reactions are presented in Figure 3. Of the six total reactions, five were found to be statistically significant (Table 2). Rubisco activase 1 was found to be up-regulated in both semigametic anthers and ovules, mirroring the expression found during microarray analysis. However, expression of Rubisco activase 2 was found to be down-regulated in both semigametic tissues, contrary to what was found in the microarray results, while there was consistent down-regulation of the Rubisco small subunit precursor in semigametic ovules in both the qRT-PCR and microarray results.

## Discussion

While there are a few microarray platforms for cotton available, we decided to use Affymetrix GeneChip Cotton Genome Array for our studies due to its technical robustness and use of multiple probes for a single gene (a total of 239,777 probe sets representing 21,854 cotton transcripts). Since 57-4 was a natural doubled haploid mutant isolated from Pima S-1, both are natural isogenic lines. A comparison between the two genotypes allows for the identification of genetic and molecular differences that may be further traced to the semigametic gene itself. For example, Zhang and Stewart (2005) reported that 57-4 had significantly reduced photosynthetic rate and chlorophyll content, shorter fiber length and higher micronaire (i.e., courser fiber), compared with Pima S-1 [8]. In this study, 284 genes in anthers and 1,864 genes in ovules were identified as being differentially expressed in the semigametic genotype 57-4 relative to Pima S-1. Although the list of common differentially expressed genes in semigametic tissues is too large to analyze individually and one of them may be the semigamy gene itself the limitation of the current microarray analysis did not allow pinpointing of the semigamy gene. However, there were several interesting genes in the group that deserve a closer examination. It should also be pointed out that 17 of the differentially expressed genes identified in our previous differential display study [12] were also identified in our current microarray analysis, further confirming the corroboration between the two gene expression technologies. Once again, it is currently unclear whether one of the genes is the semigamy gene without a completion of genetic and physical map-based cloning of the *Se *gene.

### Choline production and response to environmental stress

In plants, the metabolite choline is of vital importance because it is used to synthesize phosphatidylcholine, a major membrane lipid. Additionally, in some plant species choline is oxidized to glycine betaine, which acts as a potent osmoprotectant that confers tolerance to high salinity, drought and other environmental stresses [13]. Phosphoethanolamine N-methyltransferase is a key enzyme which catalyzes the steps necessary to convert phosphoethanolamine to phosphocholine. Recent studies have shown that silencing of phosphoethanolamine N-methyltransferase in *Arabidopsis thaliana *resulted in abnormal growth and temperature-sensitive male sterility, which was attributed to failure to produce functional pollen [13,14]. This finding bodes well with a previous differential display study comparing gene expression between semigametic 57-4 and non-semigametic Pima S-1, which also identified phosphoethanolamine N-methyltransferase as being down-regulated in semigametic tissues [12]. While down-regulation of phosphoethanolamine N-methyltransferase is likely to result in decreased choline and phosphotidylcholine levels, it may also result in lower levels of glycine betaine, which would render semigametic plants more susceptible to high soil salinity and other environmental stressors, such as reactive oxygen species. According to a previous study, some phosphoethanolamine N-methyltransferase mutants exhibited pale green leaf color when subjected to high salinity [14], which may indicate a decrease in leaf chlorophyll levels. A more recent study into the effects of salt stress on cotton revealed that the rate of photosynthesis and the activity of Rubisco decreased as salinity increased [15]. In cotton, Zhang and Stewart [8] noted that the chlorophyll content as well as the rate of photosynthesis is markedly reduced in semigametic cotton plants. Furthermore, the rate of photosynthesis, especially CO_2 _fixation, can be severely affected by reactive oxygen species, such as the superoxide radical, hydrogen peroxide, and the hydroxyl radical [16].

### Production of and response to plant hormones

Ethylene is a potent plant hormone that regulates many aspects of plant growth and development, such as fruit and flower maturation as well as other physiological effects associated with aging [17]. In cotton, production of ethylene has been shown to be one of the most significantly up-regulated biochemical pathways during fiber cell elongation and it was found that exogenously applied ethylene promoted robust fiber cell elongation, whereas its biosynthetic inhibitor L-(2-aminoethoxyvinyl)-glycine reduced fiber length [18]. The down-regulation of an ethylene responsive transcription factor identified in the semigametic tissues may have an adverse effect on ethylene production and a decrease in ethylene production in turn could result in the production of shorter, coarser fibers previously observed in the semigametic cotton 57-4 in comparison to Pima S-1 [8]. However, their relationship with respect to semigamy is currently unknown.

The hormone gibberellin has an important role in plant development and growth as well as signal transduction pathways which influence gene expression and plant morphology [19]. Gibberellic acid signaling has been shown to be a de-repressible system controlled by DELLA proteins [20]. DELLA proteins act as transcriptional modulators which repress response to gibberellins. In semigametic tissues, a gibberellic acid insensitive DELLA (DELLA-GAI) protein was found to be up-regulated in both anthers and ovules. Previously, genetically engineered apple trees containing an Arabidopsis *gai *gene exhibited a dwarfed phenotype [21] similar to the shorter statue observed in semigametic 57-4 cotton plants in comparison to Pima S-1 [8]. Gibberellic acid was also shown to induce expression of xyloglucan endotransglycosylase and expansin gene during fiber cell elongation in cotton [22]. Both xyloglucan endotransglycosylase and several expansins were found to be down-regulated in semigametic tissues, signifying that gibberellins may play some part in the semigamy phenotype.

Jasmonates are a class of plant hormone that play a key role in the regulation of reproduction, metabolism, response to abiotic stress, and defense responses against pathogens and insects [23]. Biosynthesis of jasmonates has also been shown to be of critical importance in pollen maturation and dehiscence. Previous studies have shown that knock-out mutants of allene oxide synthase, the first committed step in jasmonate synthesis result in male sterility [23,24]. Additionally, a mutant of 12-oxophytodienoate reductase was also shown to be male-sterile due to lack of jasmonic acid synthesis [25]. In semigametic anthers, allene oxide synthase was identified as down-regulated while 12-oxophytodienoate reductase was found to be up-regulated in both semigametic anthers and ovules. While both of these genes are interesting due to the fact that they can result in male sterility, the role of jasmonates in semigamy is currently unknown.

### Cytoskeletal components

Cytoskeleton plays an important critical role in plant growth and development through regulating an array of fundamental cellular processes such as cell division, cell expansion, organelle motility and vesicle trafficking. While the mechanism of movement of the sperm cells to the egg and central cell during double fertilization remains largely unknown, previous studies have shown that reorganization of the cytoskeleton may play a key role in the transport process. In studying the process of double fertilization in *Nicotiana tabacum*, Huang and Russell [26] noted dramatic changes in cytoskeletal reorganization. It has been postulated that abundant actin in the embryo sac, also called actin coronas, plays a key role in aligning the male gametes to their target cells and facilitating gametic fusion [26-28]. In our microarray analyses, several genes homologous to tubulins were found to be down-regulated in semigametic tissues and actin was found to be down-regulated in semigametic ovules but up-regulated in semigametic anthers (Table 1). The down-regulation of actin in semigametic ovules may cause the misalignment of the sperm cell and inhibition of sperm movement. Even though the function of microtubules in double fertilization is minor, their involvement in the process of semigamy in cotton is currently unknown. In addition, the mechanism by which the sperm nucleus migrates to the egg nucleus once it has penetrated the egg cell still remains enigmatic.

### Biosynthesis of gossypol

This study revealed that delta-cadinene synthase was up-regulated in both anther and ovule tissues of 57-4 as compared to these of Pima S-1. Delta-cadinene synthase is the first committed step in a multi-enzyme process leading to the production of gossypol, a polyphenolic yellow pigment produced by most cotton species that acts as a natural insecticide [29]. Gossypol is a chiral compound due to restricted rotation between the naphthalene ring systems, with the (-)-enantiomer being more biologically active than the (+)-enantiomer. Previous studies have shown that Pima cotton (*G. barbadense*) produces more of the biologically active (-)-enantiomer than the majority of other cotton species; these of the species produce more of the biologically inert (+)-enantiomer than *G. barbadense *[30,31]. The compound has great pharmacological interest due to its potential as an anti-cancer agent and for its male contraceptive abilities [29]. In human spermatozoa, gossypol was shown to inhibit the motility of sperm cells through a dose dependent mechanism [32]. Upon a closer examination, it was found that gossypol can inhibit enzymes of glycolysis and the TCA cycle, severely crippling energy metabolism and ATP production. Additional studies have shown that gossypol binds tubulin monomers non-covalently such that they cannot participate in microtubule polymerization [33]. As previously mentioned, microtubules may play a key role in transporting the sperm nucleus to the egg nucleus during karyogamy. Thus inability to form complete microtubules may inhibit karyogamy from occurring during fertilization. During our microarray analyses, several genes homologous to actin and tubulins were found to be down-regulated in semigametic tissues (Table 1). In yet another study into the effects of gossypol on a photosynthetic protist *Dunaliella bioculata*, it was noted that the motility of the flagellated protist dropped as expected, however the authors also noted a significant decline in cellular respiration and the rate of photosynthesis [34]. This finding correlates well with the observations of Zhang and Stewart [8] in semigametic cotton. Lastly, Kennedy et al. [35] observed that addition of gossypol to spermatozoa prevented the sperm from penetrating denuded hamster oocytes. Upon further analysis, they discerned that gossypol's inhibition of the autoproteolytic conversion of proacrosin to acrosin results in its contraceptive ability. This observation is particularly interesting when considering semigamy in cotton where the egg does not fuse with the sperm during fertilization. Although reproductive mechanisms in plants and animals are distinctive in many ways, there are also many common molecular processes [36]. If a system homologous to the proacrosin-acrosin system in animals were to exist in plants, the effect of gossypol may very well explain the lack of nuclear fusion between sperm and egg nuclei in semigamy. While the increased expression of delta-cadinene synthase (as it correlates with gossypol concentration) may explain many of the observed phenotypic traits associated with semigamy, a more focused study of the two active gland loci, *Gl*_*2 *_and *Gl*_*3*_, or other genes/alleles and their relationship to semigamy should be performed through gene expression studies and molecular marker analysis. Furthermore, the actual levels of gossypol, as well as the ratio of the two enantiomers, should be temporally and spatially measured in semigametic ovules and seeds relative to non-semigametic cotton.

## Conclusion

To our knowledge this is the first report using microarray technology and qRT-PCR associated with semigamy in cotton. In this study, over 2,000 differentially expressed genes associated with semigamy were identified with 127 of those genes being commonly differentially expressed in both semigametic anthers and ovules. Several important metabolic pathways affected by differentially expressed genes in the semigametic genotype have been identified and described in detail. And while these genes are not likely to be the semigamy gene itself, the effects associated with over-expressing or under-expressing their gene products do mimic phenotypic traits observed in semigametic plants. As a result, a more in-depth future analysis of their expression and regulation with respect to semigamy is necessary.

## Methods

### Plant materials and RNA isolation

Anther and ovule tissues from Pima S-1 (also designated PS-1), a normal, non-semigametic yet obsolete G. barbadense cultivar, and Pima 57-4, its naturally occurring semigametic mutant were used. Both genotypes were grown in a greenhouse in peat pots and transplanted to the field a month later. The experimental design was a paired comparison with three replicates and the plot size was single row × 40 ft long. Seeding rate was 3 seed/ft and crop production was managed as recommended locally. Anther and ovule tissues from 10 flowers were collected for each replicate of each genotype at zero days postanthesis (0 DPA) and placed in liquid nitrogen immediately and stored at -80°C. Total RNA from collected anthers and ovules was isolated using a previously described hot borate method [37]. RNA yield and quality were determined by absorbance spectra at 260 and 280 nm using a DU 530 UV/VIS spectrophotometer (Beckman Coulter, Brea, CA). After quantification, the RNA was cleaned using an RNeasy MinElute Cleanup kit (Qiagen, Valencia, CA). RNA was stored at -80°C until used.

### Microarrays and data analysis

For the microarray experiments, RNA was pooled in an equal molar ratio from the three biological replicates based on tissue and genotype. 2 mg cleaned total RNA from each of the four samples, semigametic anthers and ovules as well as non-semigametic anthers and ovules, and Affymetrix GeneChips^© ^Cotton Genome Array (Santa Clara, CA) were sent to Genome Explorations (Memphis, TN) for hybridization and preliminary data analysis. A pair-wise comparison between semigametic 57-4 and non-semigametic Pima S-1 tissues was conducted for both anther and ovule samples in order to identify differentially expressed genes. Using the Affymetrix GeneChip Operating Software the relative mean signal, detection calls, signal log ratios and change calls are independently calculated using four different algorithms for each probe set [38]. Excel files with statistically relevant up-regulated and down-regulated genes and their signal Log_2 _ratios were provided by Genome Explorations.

The sequences of differentially expressed genes identified by the microarray experiments were collected from NCBI GeneBank [39] and compared them to known sequences from Cotton Gene Index [40] using the Basic Local Alignment Search Tool (BLAST) to determine if there was any significant homology to known gene products. The results of the BLAST search were then sorted based on gene function to identify common differentially expressed genes in both semigametic anther and ovule tissue.

### Quantitative reverse transcription PCR

Nine differentially expressed genes were selected based on the microarray results (i.e., 2-12 fold changes) and putative gene functions were selected and analyzed using real-time quantitative RT-PCR. Initially, the total RNA for each sample was quantified using a DU 530 UV/VIS spectrophotometer (Beckman Coulter, Brea, CA). The total RNA was then diluted 5-fold with sterile molecular biology grade water (Promega, Madison, WI) to concentrations of 20 ng/μL, 4 ng/μL, and 800 pg/μL. Real-time PCR assays for each target gene were performed in triplicate for each of the aforementioned concentrations of total RNA, no reverse transcriptase and no template controls on a Bio-Rad iQ5 Thermal Cycler (Hercules, CA). One-step RT-PCR reactions of 20 μL volume containing 10 μL EXPRESS SYBR GreenER qPCR SuperMix Universal (Invitrogen, Carlsbad, CA), 20 nM Fluorescein reference dye (Invitrogen, Carlsbad, CA), 0.5 μL EXPRESS SuperScript Reverse Transcriptase (Invitrogen, Carlsbad, CA), 0.2 μM forward and reverse primers, 1.5 μL RNA template and 3.2 μL sterile water (Promega, Madison, WI). Reactions were run using the pre-set one-step RT-PCR with melt curve program, the cycling parameters of which were 50°C for 10 min., 95°C for 5 min., followed by 45 cycles of 95°C for 10 sec. and 60°C for 30 sec., and ending with the melt curve program. Gene expression and statistical analysis (Table 3) was performed using the Bio-Rad iQ5 optical system software utilizing relative quantification as described in the iQ5 system software instruction manual (Bio-Rad, Hercules, CA).

## Author's contributions

JZ and JMcDS conceived the study, and JZ supervised the project, revised the manuscript and finalized the paper. LRU conducted RNA isolation for microarray analysis. JC conducted the analyses and qRT-PCR, and drafted the manuscript. All authors contributed to the manuscript preparation, and read and approved the final manuscript.

## Supplementary Material

Additional file 1 **Raw microarray data for semigametic anthers**. Click here for file

Additional file 2 **Raw microarray data for semigametic ovules**. Click here for file

Additional file 3 **BLAST results for all differentially expressed genes in semigametic anthers and ovules**. Click here for file
